# The role of personality traits and engagement factors towards the use of paid stickers in personal communication messages

**DOI:** 10.12688/f1000research.122623.1

**Published:** 2022-08-09

**Authors:** Sudaporn Sawmong

**Affiliations:** 1 KMITL Business School, King Mongkut's Institute of Technology Ladkrabang, Bangkok, 10520, Thailand

**Keywords:** Personality Traits, Paid Stickers, Emoticons, Personal Messages, Emojis

## Abstract

**Background: **The research investigated the personality traits and engagement factors that influence the use of paid stickers in personal communication. The study was driven by the increasing significance of stickers such as emoticons and emojis in online dialogues. The study was hinged on the Theory of the big five personality traits; neuroticism, extraversion, openness to experience, agreeableness and conscientiousness. The study tested seven hypotheses to ascertain the effect of personality traits and engagement factors towards the utilization of paid stickers in personal communication.

**Methods:** The research applied the quantitative survey research design, where primary data was collected from respondents that had experience in using emojis and emoticons in their communications. The data was collected from respondents in Bangkok, Thailand using a structured questionnaire between May 14, 2022, and May 31, 2022.

**Results:** The results from 391 respondents indicated that conscientiousness, extraversion, openness, and neuroticism influence perceived enjoyment, while perceived enjoyment and perceived ease of use have a significant and positive influence on the intention to use paid stickers. Two elements of engagement, interactive engagement, and personal engagement were used to assess the influence of engagement parameters on the behavioral intention to use paid stickers. The intention to employ paid stickers in personal messages was found to be strongly and favorably influenced by these two engagement characteristics.

**Conclusions:** The study recommends that the creators of stickers, emoticons, and emojis should consider user personality features, sticker engagement, sticker simplicity of use, and the personal delight of users in the creative and communication process. The study concludes that perceived enjoyment and perceived ease of use have a large and favorable impact on the use of paid stickers. The study's main limitation was that it focused on one area of social media. This must be taken into account when applying the findings.

## Introduction

Emoticons and emojis are used to create stickers, which are among the instruments that may aid enhance communications. Emoji, emoticons, and stickers increase the social inclinations and complexity of online dialogues and communication. ^1^ Emoticon usage has changed with time, obtaining supplementary purposes. Widely praised for compensating for the absence of prosodic and gestural clues in textual emoticons, they portray emotion and simulate nonverbal indicators like winks and tongues hanging out that traditionally signify flirting and joking behaviors when done offline. While face-to-face communication is restricted online, it may be used to communicate emotions or transmit meanings and substitute for the lack of nonverbal indicators. ^2^ Emoticons are becoming more widely accepted as a system of punctuation for indicating utterance emotion and therefore are gaining grammatical significance.

Similarly, users' large number of emoticons when the internet was introduced has dwindled over time to only a few that are seldomly used—for example, the open-mouth laugh, the grin, the wink, the frown, and the tongue sticking out. This decrease is also in line with changes in offline language. Wang *et al.,* ^3^ point out that emojis may also do functions that emoticon can’t, like riffing on other graphics and narrating occurrences, as they can “keep a conversational link; conclude a conversational thread; amuse and establish new interpretations” (p.4). Emoji have a bigger influence on readers’ impressions of a writer's mood and devotion than emoticons, owing to their increased vividness. Emojis, which are emoticons in the shape of icons, came later. Tang and Hew ^4^ state that character emoticons and moving emoticons have become popular, known as stickers. People may use stickers to convey their feelings, ideas, and intentions more interactively. Social networking services (SNSs), personal messengers, and marketing all employ stickers.

A personal messenger may communicate with a single person or a group of individuals, but a social networking site can communicate with unlimited users. Stickers are mostly utilized as a means of communication in this environment. De Seta and Biaoqing ^5^ highlights that their usage conveys nonseriousness, frivolity, and funny purposes more abstractly. Stickers have become commonplace at the utterance-final position, where they generally appear, as markers of the user's expressive intent, such as highlighting or downplaying the power of the utterance and indicating its tone. According to Tang and Hew, ^6^ tone marking, also known as illocutionary force, is a more abstract, ‘bleached’ function than communicating emotion or nonverbal conduct. It also entails an expansion of the emoji's original meaning so that a smiling face may now convey not just pleasure or a good mood but also pragmatic connotations like honesty, tolerance, and civility. Personal messenger users consider stickers more helpful, fascinating, entertaining, simple, and casual than emoticons.

### Use of paid stickers in personal communication

Numerous businesses utilize stickers to advertise their goods to customers. They employ stickers to build their brand emblems, pique people's attention, and urge consumers to learn about their companies. ^4^ The objective of using stickers varies depending on the context in which they are used. WhatsApp, WeChat, Facebook Communication, Skype, Line, BBM, QQ Messenger, Telegram, and KakaoTalk are just a few personal messenger apps. Because several personal messengers provide emojis, emoticons, and stickers freely, the emoji and sticker industry is seldom supported. ^7^ However, companies like KakaoTalk and Line Creators have developed sticker markets where users can purchase and gift stickers.

The LINE Store is filled with free and premium stickers created by authorized groups, designers, and several LINE customers. Even though 70% of Line customers rarely purchase e-stickers, 74.5% have used sponsored e-stickers. ^8^ Customers adore these personalized e-stickers because they are free and because the design is original and vibrant. Including both a brand image and a product image in the interface style of LINE stickers unintentionally delivers sponsor imagery to consumers’ brains to be absorbed, even if it is the customer's initial interaction with the collection of stickers. Therefore, learning ways to build meaningful branded ^9^ stickers is a significant problem for marketers to enhance the efficacy of LINE sticker marketing initiatives. Iqbal ^10^ informs that in 2020, LINE made $1.51 billion in income, largely from advertising. LINE is used by approximately 224 million individuals once a month, with 86 million in Japan. In 2020, LINE users transacted about $15 billion on the service. Mobile marketing media's interactive and entertaining character may positively impact brands and customer behavior.

Similarly, from 2021, Kakao has offered a subscription option that allows users to utilize all stickers for a set monthly fee. The sticker market is growing, and the subscription service includes a sticker suggestion feature. ^11^ According to Jobst, ^12^ Kakao's yearly revenue was roughly 6.1 trillion South Korean Won in 2021, up from around 4.2 trillion earned before. Lee and Lee ^7^ clarifies out that several variables are likely to influence sticker purchases; however, only a few research studies have looked at the factors that influence sticker purchases. As a result, it's important to consider the numerous diverse elements that influence sticker purchases.

### Literature review and hypotheses

This section describes the literature review and hypotheses of the role of personality traits and engagement factors towards the use of paid stickers in personal communication, including the theory of the big five personality traits, as well as the application of the Technology Acceptance Model in investigating the research problem.

### The use of stickers, emoticons, and emojis

Several variables are likely to influence sticker purchases. However, just a few researchers have looked at the variables that influence sticker purchases. As a result, it's important to consider the numerous diverse elements that influence sticker purchases. Personality is among the variables that reveal a customer's unique characteristics, and it's often discussed regarding product and service buying and preference. ^13^ Various elements influence personal differences, including personality, developmental stage, needs, and attitudes, which are more constant over time and across contexts than other aspects. Investigating the effects of nature on individual service use patterns or preferences will help with the most long-term and stable customization. According to Dainas and Herring, ^14^ personality is widely utilized in business and psychology studies to identify attitudes and actions, and personality is a significant predictor of customer beliefs and attitudes in information management studies. When it comes to stickers, individuals buy them to use their communication in messages strongly tied to their purpose.

### Theory of big five personality traits

Many modern personality theories suggest that there are five essential dimensions to personality, designated the Big Five personality traits. The Big Five personality traits (neuroticism, extraversion, openness to experience, agreeableness, and conscientiousness) are a psychological research framework that relates to a particular propensity expressed through intra-psychic characteristics. ^15^^–^^16^ The traits are defined as natural inclinations; the actions they incline one to vary across tasks, social environments, and timeframes, but they are all consistent with the trait. Because the items are mainly context-invariant, the assessments of the five qualities are frequently decontextualized. ^15^ Because it may reflect many systems of personality description in a shared framework, the Big Five personality traits perform an integrative function. ^17^ These five characteristics have been studied and evolved, and while each has received a lot of attention, scholars have not always agreed on how to define the different traits. The five personality trait tests determine where an individual falls in the range of each of the five qualities. Each attribute assesses a distinct facet of human personality:

*Neuroticism*

Anxiety, self-consciousness, aggression, and mental illness are all symptoms of neuroticism. ^15^ The outcomes of neuroticism research vary by field. First, those with high neuroticism seem to have a pessimistic view of technology's utility. Şahin *et al.,* ^16^ infers that people with high neuroticism see technological growth as dangerous and unpleasant, so neuroticism negatively connects with technology's perceived utility. In a virtual reality team setting, neuroticism is positively connected with technological communication anxiety, so an individual with high neuroticism is concerned about prospective obligations and events. ^17^ Furthermore, using new or old technology in novel ways creates circumstances in which future effects are unknown. However, Goreis and Voracek ^18^ are of the opinion that individuals exhibit a distinct pattern while participating in online interactions, for instance, on social networking sites or shopping, instead of experimenting with new technologies or employing cooperative technologies. Individuals with high neuroticism seek knowledge, network, and make their products available on the internet to escape the strain of face-to-face engagement. ^19^ The utilization of social services is positively associated with neuroticism. Individuals with a high degree of neuroticism are more likely to begin blogging as a digital expression. Finally, individuals with high neuroticism do not appear to be resistant to new technologies or features.

*Extraversion*

Social, outgoing, active, and warm interpersonal interactions define extraversion. ^20^ Buecker *et al.,* ^20^ further explains that energy, spontaneity, dominance, and sociability are more extroverted, while introverted people are regarded as sluggish, restrained, contemplative, and silent. According to previous studies, those with high extroversion are more active in adopting technology. Extraversion is linked to a desire to utilize a software platform constructively. ^18^ Extraversion had a favorable influence on behavioral intention. The study by Şahin *et al.,* ^16^ on the link between instructors’ personalities and their behavioral intention. Extraversion predicts the perceived utility of a virtual reality team and the desire to employ one. In the literature, though, inconsistent outcomes have been documented. Extraversion concerns external sources; hence, it entails superior performance while engaging with people or accomplishing group activities. ^21^ Since it was passive information technology, such individuals could not connect with others via the learning system, resulting in a negative association. From this perspective, it may be inferred that talking with people through SNSs or messengers is significantly connected with extraversion. Furthermore, extraverted persons love social attention and thrill-seeking hobbies. They also have a low level of emotional arousal.

*Openness to experience*

Nikčević *et al.,* ^19^ state that curiosity, inventiveness, inquisitiveness, and aesthetic sensitivity are all characteristics of openness to experience. People that are open to new experiences and beliefs actively seek them out. Openness studies have shown a variety of outcomes. According to several studies, openness is linked to a desire to utilize technology. Openness, for instance, is a personality quality that impacts the desire to work with a virtual reality group. Openness is linked to a good desire to utilize technology. On the other hand, openness had no bearing on the desire to utilize technology; it only impacted perceived ease of use (PEU). Furthermore, openness has little impact on the use of mobile applications. Şahin *et al.,* ^16^ claimed that the mobile applications they utilized in their research diminished openness since many people widely adopted them. A more open person is more inclined to take chances to succeed. As a result, if many individuals have already adopted a service or technology, it is no longer a risky position. Most individuals use stickers and messengers daily, and openness is not anticipated to take a motivational role in using or purchasing stickers.

*Agreeableness*

According to Buecker *et al.*, ^20^ kindness, helpfulness, being thoughtful, and being altruistic toward others are characteristics of an agreeable personality. Agreeableness is linked to interpersonal connections. Individuals that are agreeable are socially oriented and want a sense of belonging. Stajkovic *et al.,* ^15^ adds that agreeable individuals want social value when utilizing social networking sites; therefore, pleasant individuals are socially driven toward assisting and collaborating with others. Numerous research has shown a link between agreeableness and perceived usefulness when utilizing technology. ^21^ When working with new technology, friendly individuals are more responsive and collaborative, and they strive to find the good elements. Furthermore, if technology facilitates cooperation in completing tasks and job completion, it will be significantly linked to agreeability. ^22^ Individuals with a high agreeableness use stickers to show pleasant feelings toward others. Emojis representing blushing cheeks are associated with agreeableness because blushing is a signal that promotes favorable social relationships. As a result, persons with high agreeableness will want to participate in more emotional interactions, and stickers are an excellent tool. As a result, it was expected that persons with a high agreeability would utilize stickers to convey their feelings better and demonstrate closeness.

*Conscientiousness*

People who have high conscientiousness follow the rules, exercise self-control, and put in a lot of effort. ^23^ The link between conscientiousness and social media usage has been studied in certain research. More conscientious people are less likely to actively engage in social media and those who are more conscientious show more remorse when they share improper content. ^24^ When they use SNSs, they look for content that has monetary worth. Individuals with strong conscientiousness are tight with themselves and encourage online learning activities while rejecting online pleasure pursuits. They shun leisure applications since they are ineffective or bothersome for mobile apps. Individuals use stickers for various purposes, but the fundamental goal of personal messengers is to interact. Individuals with high conscientiousness will utilize stickers for this reason if stickers increase the productivity of the discussion. The phrase complement motive is linked to precise communication and emphasizing delivery, which is important for enhancing conversation performance. ^18^ Individuals with high conscientiousness, according to, ^24^ are strict with themselves and prefer online learning activities while rejecting online pleasure pursuits. As a result, we hypothesized that a high level of conscientiousness would be connected with a desire to complete sentences.

*Hypothesis 1*
***(H1)**:* The big five personality traits (at least three of which) have a significant and positive influence on perceived enjoyment of paid stickers.

*Hypothesis 2*
***(H2)*** *:* The big five personality traits (at least three of which) have a significant and positive influence on the perceived usefulness of paid stickers.

### Personal engagement and interactive engagement effects on behavioral intention to use

Personal and interactive engagements have been investigated in associated academic areas like sociology, psychology, and organizational behavior, but it has not been emphasized lately in the marketing sector. Researchers in marketing have begun to look at the topic, particularly in the service and mobile settings. ^25^ Zainuddin *et al.,* ^26^ reiterates that consumer engagement is widely thought to depend on connections between the engagement subject and the engagement object, even though studies have yet to agree on consumer engagement. According to several academics, interactive engagement has behavioral, cognitive, and emotional elements. Liu *et al.,* ^13^ specifically developed a measuring instrument for personal messenger interactive engagement, referring to these three characteristics as cognitive processing, attachment, and activation. These dimensions are theoretically, and an empirically distinct notion, which implies their nomological networks, may vary, and their determinants, consequences, and moderators may or may not be the same. ^25^ Furthermore, they play distinct functions in different circumstances, resulting in variable degrees of participation.

*Hypothesis 3*
***(H3):*** Interactive engagement has a significant and positive influence on the behavioral intention to use paid stickers

*Hypothesis 4*
***(H4):*** Personal engagement has a significant and positive influence on the behavioral intention to use paid stickers

### Technology acceptance model (TAM)

The technology acceptance model (TAM) originated with a curiosity about the elements that influence people's acceptance or rejection of information technology. ^27^ The TAM has been investigated in terms of motivation. Salloum *et al.,* ^28^ state that extrinsic motivation is a kind of it, and perceived usefulness and proposed an integrated technology adoption model that includes intrinsic motivation via pleasure. According to research, employing perceived pleasure to quantify intrinsic motivation, extrinsic and intrinsic incentives promote sustained SNS use. ^29^ Many researchers have tried to explain motivational aspects using the TAM, although the questions posed in this research deal with abstract dimensions. Estriegana *et al.,* ^30^ looked at the distinctiveness of learning and the incentives linked with environmental aspects on the internet, indicating that particular motivations are more contextual than generic. The use of stickers in personal messengers has its own set of peculiarities. They are mainly available online, are simple to use, and are handy. The latter is a common feature of social networking sites.

*Perceived enjoyment effect on behavioral intention*

Individuals’ intrinsic drive to utilize a system is perceived as delightful, ^31^ Sukendro *et al.,* ^31^ stated that consumers generally accept emerging technologies and techniques since they may bring intrinsic perks like entertainment, enjoyment, etc. A few earlier studies have only studied the association between perceived enjoyment and behavioral intentions. ^32^ Chao ^33^ states that enthusiasm, pleasure, and other factors of perceived enjoyment have a substantial impact on behavioral intention. Users who have a positive attitude about new technology will be more likely to utilize the new application. The time of usage is connected to pleasure or perceived enjoyment. Pleasure and excitement will raise anticipation, hence affecting behavioral intention.

*Hypothesis 5*
***(H5):*** Perceived enjoyment has a significant and positive influence on the behavioral intention to use paid stickers

*Perceived ease of use effect on behavioral intention*

‘Ease of use’ is described as a person's view that using new technology relieves them of strain. ^34^ Sun and Gao ^35^ suggested that perceived ease of use is a system that is considered simple to grasp, analyze, or operate, causing buyers to plan to purchase online. Lin *et al.,* ^36^ reiterates that consumers’ perceptions of the ease of use and adaptability of stickers’ impact customers’ motives towards behavioral intention. Perceived ease of use is related to behavioral intention.

*Hypothesis 6*
***(H6):*** Perceived ease of use has a significant and positive influence on the behavioral intention to use paid stickers

*Perceived usefulness effect on behavioral intention*

The extent to which an individual feels that implementing a strategic approach would help them achieve more success is perceived usefulness. ^37^ Chao ^33^ defines perceived usefulness as how humans feel that employing a given system would increase their work performance. When it comes to perceived usefulness, people typically inquire about how technology helps them increase their efficiency, profitability, and efficacy. ^35^ Customers’ perceptions of stickers’ usefulness are regarded as an innovative feature that aids in developing more efficient methods for measuring consumers’ behavioral engagement.

*Hypothesis 7*
***(H7):*** Perceived usefulness has a significant and positive influence on the behavioral intention to use paid stickers

## Methods

### Ethical statement

This study was approved by the Research Ethics Committee of King Mongkut's Institute of Technology Ladkrabang, Thailand with study number EC-KMITL_65_077. The Ethics Committee granted the study an exemption waiver in line with the Declaration of Helsinki, ICH Guidelines for Good Clinical Practice, and other international guidelines for human research protection. We confirm that all respondents gave their informed consent voluntarily. The questionnaire did not include any information that might be used to identify respondents. They could also refuse to answer any question that they felt invaded their privacy. Participants were promised that any freely submitted information in the course of this study would be treated confidentially, including any information that could reveal their identity. Written informed consent was obtained in the first stage of the questionnaire section before respondents are exposed to the questionnaire questions proper.

### Participant collection

The study was a survey research design focused on investigating the role of personality traits and engagement factors in using paid stickers in personal communication such as in Messengers. The research relied on primary data collected from a representative sample of the respondents. The study population was the people who use stickers such as emoticons and emojis in their online conversations. Therefore, the population of the study was quite large. The people in Bangkok city were considered suitable for sampling to determine the sample size. The questionnaire was developed in English and hosted online using Google Forms; a campaign was run requesting people take part in the survey on social media platforms including Facebook, LINE, Instagram, Twitter, and Whatsapp. Users on these platforms that fit the study criteria were encouraged to complete the survey. The target sample size was 600 respondents, and 473 people answered the questionnaire. Before respondents are taken to the main questionnaire, they gave their written consent to participate in the study with the undertaken that their data will be kept confidential, and that the questions do not involve a personally identifying questions. Respondents were also informed that they had the option of exiting the study at any point they are not comfortable with the nature of the questions being asked. After cleaning the data from respondents, including removing those with incomplete answers or illegible responses, 391 responses were considered suitable for data extraction for the study. The survey was conducted between May 14, 2022, and May 31, 2022.

### Questionnaire

The questionnaire was divided into two main sections; the first section was used to produce information about the demographic data of the respondents, while the second section was used to elicit responses to the research hypotheses. ^64^ The first section of the demographic data covered the gender, age, education, employment and income of the respondents and their use of paid stickers. The section had seven questions, while the second section had 54 questions covering the study hypotheses. Extraversion, agreeableness, conscientiousness, neuroticism, and openness to experience had six questions each, while perceived ease of use, perceived usefulness, perceived enjoyment, purchase intention, and interactive engagement had four questions each. The scales and the variables are summarized in Table 1. Prior to the main study survey, a pilot study was undertaken to test the reliability of the research instrument by administering the questionnaire to 30 respondents from May 14–16, 2022. The pilot study was hosted using Google Forms and shared with participants using Facebook and the Line app. Once the target sample (30) was achieved, the pilot study was discontinued. The final survey did not contain these initial respondents. This made it possible for the author to evaluate the appropriateness and readability of each questionnaire item. The pilot study was consistent with the intent of the research thus no changes were required to the questionnaire.

**Table 1.** Latent variables, scales, and sources.

Latent variables	Scales	Sources
Extraversion	I feel comfortable around people.	^38^^–^^39^
	I keep in the background.	
	I start conversations.	
	I have little to say.	
	I talk to many different people at parties.	
	I do not like to draw attention to myself.	
Agreeableness	I insult people.	^40^^–^^41^
	I sympathize with others' feelings.	
	I am not interested in other people's problems.	
	I have a soft heart.	
	I am not really interested in others.	
	I take time out for others.	
Conscientiousness	I pay attention to details.	^42^^–^^44^
	I make a mess of things.	
	I get chores done right away.	
	I often forget to put things back in their proper place.	
	I like order.	
	I shirk my duties.	
Neuroticism	I worry about things.	^45^^–^^46^
	I seldom feel blue.	
	I am easily disturbed.	
	I get upset easily.	
	I change my mood a lot.	
	I have frequent mood swings.	
Openness to experience	I have excellent ideas.	^40^^,^^45^
	I do not have a good imagination.	
	I am quick to understand things.	
	I use difficult words.	
	I spend time reflecting on things.	
	I am full of ideas.	
Perceived ease of use	Learning to use emoticons is easy for me.	^44^^,^^47^
	I find it easy to use emoticons when messaging	
	It would be easy for me to become skillful in using emoticons.	
	My interaction using emoticons is clear and understandable.	
Perceived usefulness	Using emoticons improves my conversation in the messenger.	^48^^–^^49^
	Using emoticons helps me express my emotions accurately	
	Using emoticons enables me to do my message more efficiently.	
	Using emoticons increases my productivity.	
Perceived enjoyment	Using emoticons makes me feel good.	^47^^,^^50^^–^^51^
	Using emoticons is enjoyable.	
	Using emoticons if fun while chatting	
	Using emoticons is interesting.	
Purchase intention	I find purchasing emoticons to be worthwhile.	^50^^–^^52^
	I will frequently purchase emoticons in the future.	
	I will strongly recommend others to purchase emoticons.	
	I will recommend others to use emoticons while chatting	
Interactive Engagement	I feel psychologically connected to others while using emoticons	^53^
	I have pleasurable emotional state while using emoticons during interactions	
	There is a sense of pride to be able to use emoticons during conversation	
	Attitudes and expressions are displayed well using emoticons	
Personal Engagement	I am able to express myself well while using emoticons	
	I expresses my emotions well using emoticons during conversation	^54^^–^^55^
	There is a sense of empowerment from engagement resulting from use of emoticons	
	Using emoticons has a fulfilling and positive state of mind	

Source: Adapted from Kang *et al.,.* ^56^

### Data collection and processing

The data was collected using a 5-point Likert scale on a questionnaire. his contained scales ranging from 1 to 5, with 1 indicating ‘strongly disagree’ and 5 indicating ‘strongly agree’. ^63^^–^^64^ The researcher made certain that the questionnaire did not include any options that could divulge personal or identifying information. The study participants stayed anonymous as a result of this. Several strategies were used to analyze the data. The first phase was data cleaning, which included eliminating missing data, identifying and deleting outliers, and removing any numbers that did not appear to be consistent with the rest of the data. This was followed by descriptive statistics, which entails computing the characteristics of the data's variables, such as mean, mode, median, standard deviation, percentiles, skewness, kurtosis, and maximum and minimum values. Normality tests, correlation descriptive statistics, validity and reliability testing, and confirmatory factor analysis were among the diagnostic tests used. Utilizing AMOS version 26, Structural Equation Modeling (SEM) was used to evaluate the study's hypotheses.

### Variables

**Figure 1.**
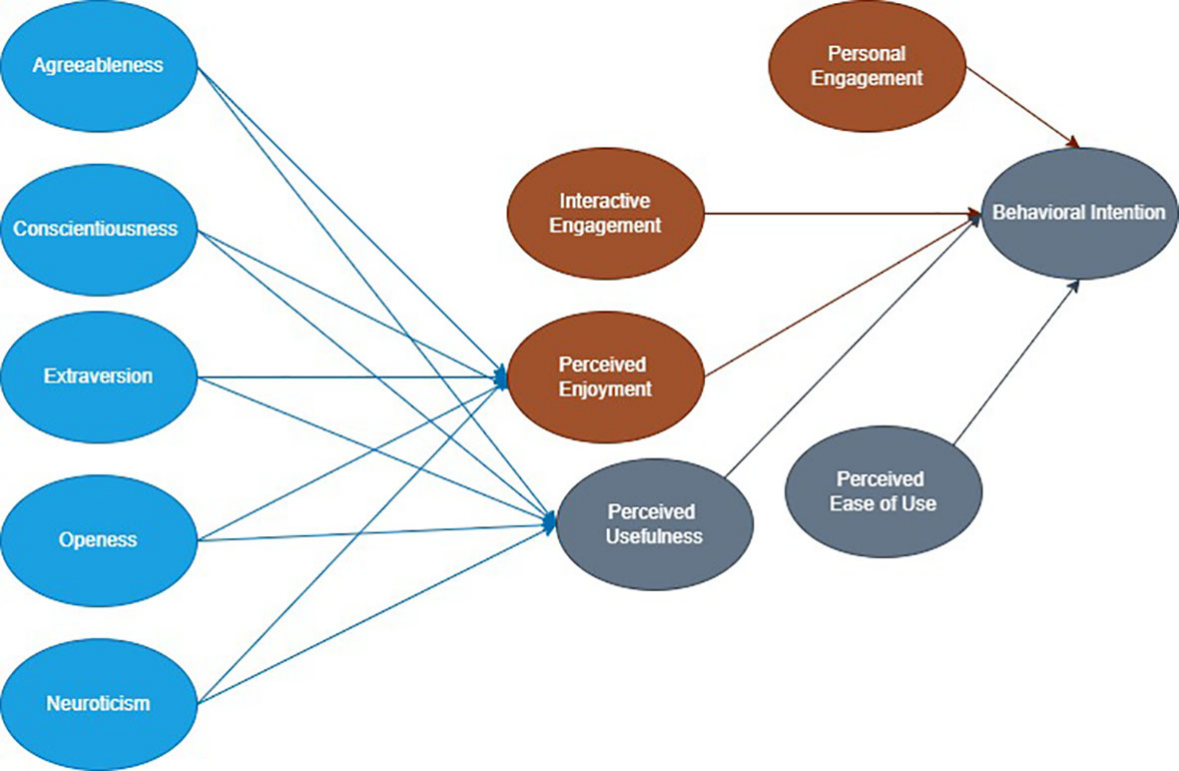
The conceptual framework of paid stickers in personal communication messages.

The study variables were developed from a critical literature review and applicable theoretical frameworks. ^56^ The personal traits used in the study were obtained from the big five personality traits, which include neuroticism, ^45^^–^^46^ extraversion, ^38^^–^^39^ openness to experience, ^40^^–^^45^ agreeableness, ^40^^–^^41^ and conscientiousness. ^42^^–^^45^ The engagement factors were represented by personal engagement and interactive engagement. The Technology Acceptance Model provided four additional variables: perceived enjoyment, ^47^^,^^50^^–^^51^ perceived ease of use, ^44^^,^^47^ perceived usefulness, ^48^^–^^49^ and behavioral intention to use. ^50^^–^^52^ Among these variables, perceived enjoyment and usefulness were the mediating variables; behavioral intention was a dependent variable, while others were independent variables, this is summarized in Table 1. The applied conceptual framework is presented in Figure 1.

Various techniques were adopted in the process of data analysis. The first was evaluating the model's suitability and fitness of the study variables in the proposed conceptual framework. This was done using reliability and validity techniques, including Composite Reliability (CR), Average Variance Extracted (AVE), Cronbach's alpha, and factor loadings. The measurement of the proposed research model was conducted using convergent validity, discriminant validity, and internal consistency. For composite reliability, the indicator threshold was supposed to be above or equal to 0.7 based on the recommendations of Hair *et al.,.* ^57^ The model's fitness was evaluated using confirmatory factor analysis (CFA) with various indicted being tested. The Partial Least Square Structural Equation Modeling (PLS-SEM) was utilized in the analysis of the proposed hypothesis.

### Trustworthiness and credibility of the research data

In research projects, establishing trustworthiness entails establishing the reliability of the research process. Credibility refers to the qualitative researcher's belief in the accuracy of the study's findings. To be believed, the researchers had to show that data collection and analysis were done in a specific, constant, and detailed method by documenting, standardizing, and revealing the techniques of analysis in sufficient detail to allow the reader to evaluate whether the process was plausible. In the sections above, the entire research process has been explained in detail including, method of data collection, time frame of data collection, method of analysis, and the triangulation method used in the analysis to robustly analyze the data,

## Results

The first analysis was to evaluate the demographic characteristics of the respondents. The results indicated in Table 2 show for the gender, females were the majority (54%) while males were the minority (46%). Considering the age variable, the majority age group was 21 – 30 years, followed by 31 – 40 years then 18 – 20 years, and then the least were over than 60+ years. Most of the respondents had a bachelor’s degree (34%) while the least represented were those with postgraduate degrees (17%) In terms of occupation, about 27% of the respondents were company employees, while the unemployed accounted for the least numbber of respondents with about 11%. When assessing the income of the respondents, those earning between 20,000 Bahts and 30,000 Bahts constituted over 24% of the respondents while those earning less than 10,000 Bahts were the least with about 14% of the respondents. For the ‘how often the respondents used the sticker’ variable, the majority was 11 – 20 times, followed by those who have used stickers and emoticons 21 – 30 times. For the frequency of purchase of the sticker, minority respondents indicated 11 – 20 times, followed by 21 – 30 times and the least was 40+ times.

**Table 2.** Demographic characteristics of the respondents.

Variable		Frequency	Percent
Gender	Male	179	46%
	Female	212	54%
Age	18-20 Years	61	15.6%
	21-30 Years	110	28.13%
	31-40 Years	76	19.44%
	41-50 Years	54	13.81%
	51-60 Years	57	14.58%
	Older than 60 Years	33	8.44%
Education	Junior High Scool or Lower	87	22.25%
	High School/Diploma	102	26.09%
	Bachelor Degree	134	34.27%
	Postgraduate or Higher	68	17.39%
Occupation	Student	83	21.23%
	Government Officer	64	16.37%
	Company Employee	109	27.88%
	Self-Employed	89	22.76%
	Unemployed	46	11.76%
	Others	0	0%
Monthly Income	Less than or Equal to 10,000 Baht	58	14.83%
	More than 10,000 Baht - 20,000 Baht	79	20.20%
	More than 20,000 Baht - 30,000 Baht	95	24.30%
	More than 30,000 Baht - 40,000 Baht	91	23.27%
	More than 40,000 Baht	68	17.40%
Often use stickers	1-10 times	87	22.25%
	11-20 times	114	29.16%
	21-30 times	89	22.76%
	31-40 times	66	16.88%
	40+ times	35	8.95%
Often purchase stickers	1-10 times	94	24.04%
	11-20 times	109	27.88%
	21-30 times	101	25.83%
	31-40 times	52	13.30%
	40+ times	35	8.95%

### Measurement Model Assessment

The measurement of the proposed research model was conducted using convergent validity, discriminant validity, and internal consistency. For composite reliability, the indicators threshold was supposed to be above or equal to 0.7. ^57^ From the presented results, it was found that the composite reliability results ranged from 0.701 to 0.922 which were all within the required threshold. The internal consistency of the measurement model was evaluated using Cronbach's alpha coefficients and the average variance extracted (AVE). The required threshold for Cronbach's alpha is at least 7.0 which indicate to be acceptable, good, or excellent. The results for Cronbach's alpha ranged from 0.729 to 0.937 which confirms that the indicators were within the required threshold. ^57^^–^^58^ Considering the AVE, the required threshold, according to Ramaya *et al.,* ^59^ and Hair *et al.,* ^57^ should be 0.5 or greater. From the results, the AVE ranged from 0.553 to 0.762, which satisfied the required threshold. These findings confirmed that the measurement model’s internal consistency, validity, and reliability were satisfactory and within the required threshold.

**Figure 2.**
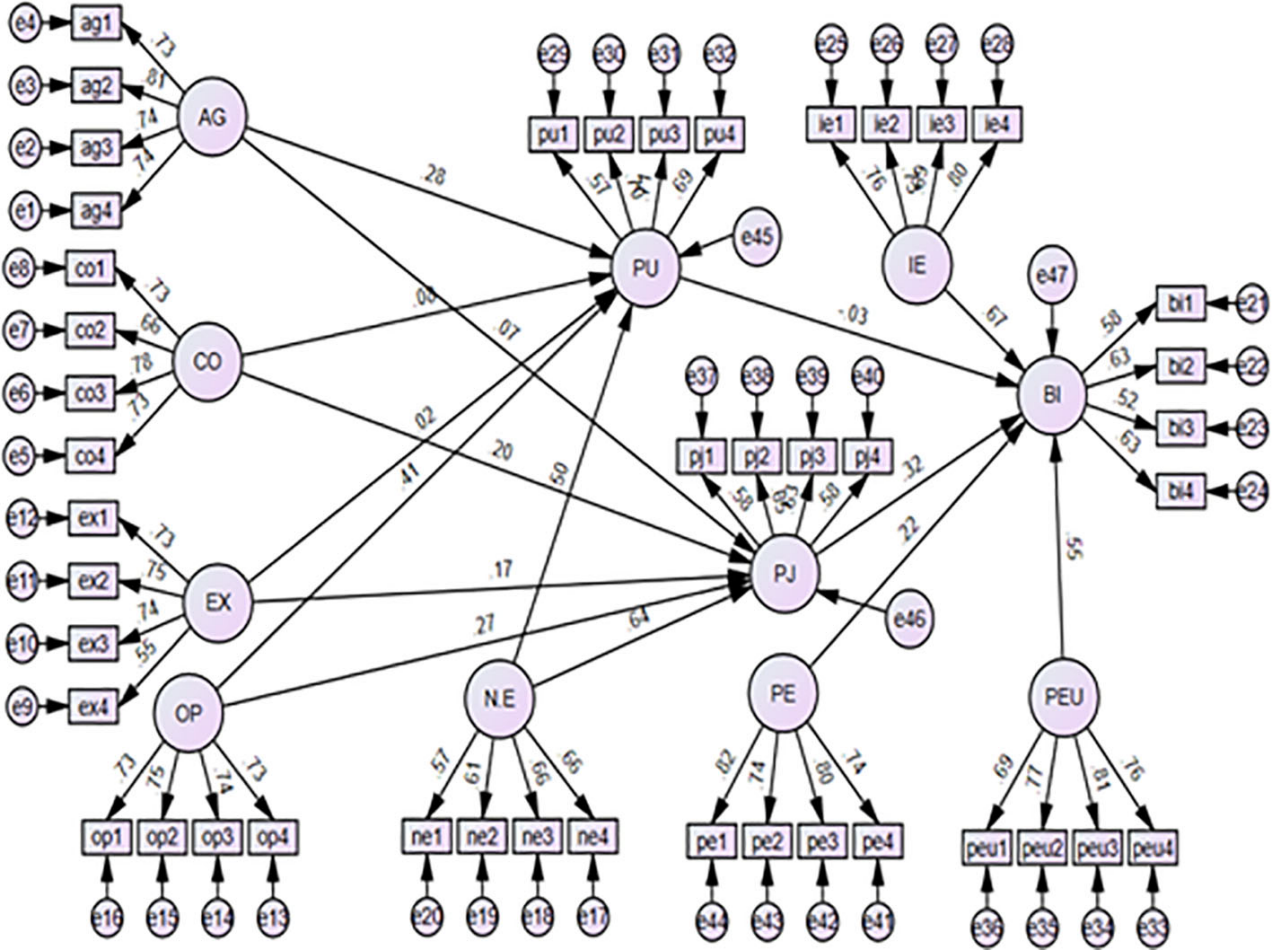
Confirmatory factor analysis model.

The fitness of the model was also evaluated by checking the fit indices of the model which demonstrated the goodness of fit of the proposed model. From the goodness of fit tests done using confirmatory factor analysis, it was found that GFI = 0.942, NFI = 0.951, CFI = 0.963, TLI = 0.968, RFI = 0.945, AGFI = 0.863, RMR = 0.048, RMSEA.096, SRMR = 0.0456, and the Chi-square/df = 2.901 as shown in Figure 2 and Table 3. These figures depicted a satisfactory fitness based on the thresholds recommended by. ^60^^–^^62^

**Table 3.** Confirmatory factor analysis (CFA) and model evaluation.

Paths		Factor loadings	CR	AVE	Cronbach’s alpha
Agreeableness (AG)			0.909	0.577	0.882
AG	ag1	0.744			
AG	ag2	0.814			
AG	ag3	0.731			
AG	ag4	0.735			
Behavioral intention (BI)			0.801	0.628	0.782
BI	bi1	0.753			
BI	bi2	0.791			
BI	bi3	0.71			
BI	bi4	0.788			
Conscientiousness (CO)			0.949	0.723	0.826
CO	co1	0.729			
CO	co2	0.702			
CO	co3	0.764			
CO	co4	0.707			
Extraversion (EX)			0.701	0.629	0.924
EX	ex1	0.732			
EX	ex2	0.698			
EX	ex3	0.702			
EX	ex4	0.646			
Interactive engagement (IE)			0.907	0.600	0,729
IE	ie1	0.737			
IE	ie2	0.71			
IE	ie3	0.748			
IE	ie4	0.776			
Neuroticism (NE)			0.903	0.762	0.927
N.E	ne1	0.739			
N.E	ne2	0.491			
N.E	ne3	0.532			
N.E	ne4	0.644			
Openness (OP)			0.816	0.629	0.738
OP	op1	0.742			
OP	op2	0.744			
OP	op3	0.727			
OP	op4	0.745			
Personal engagement (PE)			0.922	0.607	0.783
PE	pe1	0.806			
PE	pe2	0.751			
PE	pe3	0.791			
PE	pe4	0.758			
Perceived ease of use (PEU)			0.862	0.558	0.937
PEU	peu1	0.73			
PEU	peu2	0.773			
PEU	peu3	0.775			
PEU	peu4	0.757			
Perceived enjoyment (PE)			0.905	0.553	0.863
PJ	pj1	0.672			
PJ	pj2	0.71			
PJ	pj3	0.725			
PJ	pj4	0.682			
Perceived usefulness (PU)			0.795	0.610	0.826
PU	pu1	0.666			
PU	pu2	0.76			
PU	pu3	0.782			
PU	pu4	0.776			

### Hypothesis evaluation

The goal of this study was to see how personality traits and engagement factors influenced the use of paid stickers in personal messages, such as emoticons and emojis. The results tabulated in Table 4 indicated that four of the big five personality traits significantly and positively influence perceived enjoyment (conscientiousness β = 0.144, p<0.000; extraversion β = 0.170, p<0.000; openness β = 0.205, p<0.000; neuroticism β = 0.544, p<0.000). This confirmed H1 that big five personality traits (at least three) have a significant and positive influence on perceived enjoyment of paid stickers. The results also indicated that three of the big five personality traits significantly and positively influence perceived usefulness (agreeableness β = 0.198, p=0.000; openness β = 0.275, p<0.000; neuroticism β = 0.380, p<0.000). As a result, H2 was supported that the big five personality traits (at least three) have a significant and positive influence on the perceived usefulness of paid stickers. The path coefficient between interactive engagement and the behavioral intention was positive and significant (β = 0.415, p<0.000) confirming H3 that interactive engagement has a significant and positive influence on the behavioral intention to use paid stickers.

**Table 4.** Results of the study for each hypotheses outlined.

Hypothesis	Paths			Estimate	S.E.	C.R.	P
**H1**	AG	➔	PJ	.056	.043	1.326	.185
	CO	➔	PJ	.144	.043	3.364	***
	EX	➔	PJ	.170	.060	2.845	.004
	OP	➔	PJ	.205	.045	4.529	***
	N.E	➔	PJ	.544	.074	7.352	***
**H2**	N.E	➔	PU	.380	.057	6.616	***
	OP	➔	PU	.275	.044	6.270	***
	EX	➔	PU	.019	.049	.384	.701
	CO	➔	PU	.054	.036	1.518	.129
	AG	➔	PU	.198	.041	4.845	***
**H3**	IE	➔	BI	.415	.045	9.185	***
**H4**	PE	➔	BI	.143	.032	4.510	***
**H5**	PJ	➔	BI	.262	.058	4.533	***
**H6**	PEU	➔	BI	.326	.039	8.468	***
**H7**	PU	➔	BI	-.032	.055	-.577	.564

AG = agreeableness, CO = conscientiousness, EX = extraversion, OP = openness, NE = neuroticism, IE = interactive engagement, PE = personal engagement, PU = perceived usefulness, PJ = perceived enjoyment, BI = behavioral intention, PEU = perceived ease of use

The path coefficient between personal engagement and the behavioral intention was positive and significant (β = 0.143, p<0.000) confirming H4 that personal engagement has a significant and positive influence on the behavioral intention to use paid stickers. The path coefficient between perceived enjoyment and the behavioral intention was positive and significant (β = 0.262, p<0.000) confirming H5 that perceived enjoyment has a significant and positive influence on the behavioral intention to use paid stickers. The path coefficient between perceived ease of use and the behavioral intention was positive and significant (β = 0.326, p<0.000) confirming H6 that perceived ease of use has a significant and positive influence on the behavioral intention to use paid stickers.

## Discussion

The objective of this research was to investigate the role of personality traits and engagement factors in influencing the use of purchased or paid-for stickers in personal communication messages. To evaluate the personality traits, the big five personality traits were used – neuroticism, extraversion, openness to experience, agreeableness, and conscientiousness. The results indicated that these factors were significant influencers of the perceived enjoyment and perceived usefulness of the use of paid-for stickers. Considering the specific aspects, conscientiousness, extraversion, openness, and neuroticism influence perceived enjoyment when using paid stickers in personal messages. These findings echo that of Ali ^24^ who indicated that individuals with strong conscientiousness are tight with themselves and encourage online learning activities while rejecting online pleasure pursuits. Nikčević *et al.,* ^19^ observed that openness is linked to a desire to utilize technology. According to Goreis and Voracek, ^18^ those with high extraversion are more active in adopting technology as well as the desire to utilize a software platform constructively.

In a similar breath, agreeableness, openness, and neuroticism have a significant influence on the perceived usefulness of paid stickers in personal messages. These findings are in line with that of Buecker *et al.,,* ^20^ who indicated that individuals that are agreeable are socially oriented and want a sense of belonging. Agreeable individuals want social value when utilizing social networking sites; therefore, pleasant individuals are socially driven toward assisting and collaborating with others. Similarly, Goreis and Voracek ^18^ pointed out that individuals with high neuroticism seek knowledge, network, and make their products available on the internet to escape the strain of face-to-face engagement.

The effects of engagement factors on the behavioral intention to use paid stickers on personal communication messages were evaluated using two aspects, interactive engagement, and personal engagement. These two engagement factors were found to significantly and positively influence the intention to use paid stickers in personal messages. This implied that the engagement aspects such as psychological connection, pleasurable emotional state, sense of pride, ability to express oneself, express emotions, and sense of empowerment associated with paid stickers in personal messages increases their uses. Liu *et al.,* ^13^ agreed with these conclusions, stating that personal comunication messages interactive engagement is associated with connections between the engagement subject and the engagement object, even though studies have yet to agree on consumer engagement.

In addition, the technology acceptance model factors were used to evaluate their influence on the behavioral intention to use paid stickers in personal messages. The factors used were perceived enjoyment, perceived usefulness, and perceived ease of use. The results indicated that perceived enjoyment and perceived ease of use have a significant and positive influence on the intention to use paid stickers such as emoticons and emojis in personal messages. It implied that enjoyment aspects such as feeling good, enjoyable, and interesting feelings while using stickers are important in their use. As well, as perceived ease of use aspects such as ease of using, ability to become skillful, and ability to clearly understand if important for the behavior towards adopting paid stickers in personal messages. These findings were supported by Lin *et al.,,* ^36^ who claimed that consumers’ perceptions of the ease of use and adaptability of stickers impact customers’ motives towards behavioral intention. Additionally, Chao ^33^ stated that enthusiasm, pleasure, and other factors of perceived enjoyment have a substantial impact on behavioral intention to adopt and use the technology. When the users perceive the technology as enjoyable, they tend to use it more often.

### Implications and contributions

This study has both theoretical and managerial implications. Considering the theoretical implications, the study has contributed significantly to the available literature regarding the adoption and use of paid stickers in personal messages. As mentioned in the literature, there is a dearth of scholarship about the issue under examination in the study. Another theoretical contribution is that the study has proposed a new conceptual framework, which could be considered in future studies. The conceptual framework is a hybrid of three models, the Technology Acceptance Model, the Big Five Personality Traits Model, and the Personal Engagement Model. The three models were combined to evaluate how their inherent factors influence the behavior intention to use paid stickers in personal messages. Considering the managerial implications, several recommendations could be made for the people in the business of developing and selling stickers such as emojis and emoticons; the study recommends that the five personality traits should be considered in developing stickers, as they influence user enjoyment, which is a critical factor in the use of stickers such as emojis and emoticons. Secondly, developers should consider the ability of the stickers to have personal engagement and interactive engagement, as this would enhance the connection between the persons communicating. Additionally, enjoyment factors such as feeling good, enjoyable, and interesting feelings while using stickers are important in their use, as well as ease of using, ability to become skillful, and ability to clearly understand.

## Conclusions

From the study, several conclusions could be developed, regarding the role of personality traits and engagement factors in the use of paid stickers in personal communication messages. The first conclusion to mention is that this developed and adopted an extended study model, which combined three theories – personality trait theory under the big five personality traits, the TAM theory, and the personal engagement factors. The framework led to insightful results regarding the use of paid stickers on personal messages. The study concludes that four personality traits - conscientiousness, extraversion, openness, and neuroticism - influence perceived enjoyment. Additionally, agreeableness, openness, and neuroticism have a significant influence on the perceived usefulness of paid stickers in personal messages. Considering the personal engagement factors, this research concludes that both interactive engagement and personal engagement are important factors in determining the behavior intention to use paid stickers in personal messages. The research also concludes that perceived enjoyment and perceived ease of use have a significant and positive influence on the intention to use paid stickers such as emoticons and emojis in personal messages. The limitation of the study was that it specifically focused on one aspect of social media, the paid stickers such as emoticons and emojis. This fact needs to be recognized in the application of the results. Another limitation is that the study was carried out in Thailand, specifically in Bangkok city. This brought about the geographical limitation which should be considered in the application of the results.

## Data availability

### Underlying data

Figshare: Paid sticker article datasets. https://doi.org/10.6084/m9.figshare.19964480.v5. ^63^

The project contains the following underlying data:

• Paid Sticker Article Datasets.xls. (Anonymised raw data responses to the survey).

### Extended data

Figshare: Paid sticker article datasets. https://doi.org/10.6084/m9.figshare.20217719.v3. ^64^

This project contains the following extended data:

• Survey form.pdf. (Paid sticker questionnaire used in this study).

Data are available under the terms of the Creative Commons Attribution 4.0 International license (CC-BY 4.0).
