# Supplementary material for: The role of personality traits and engagement factors towards the use of paid stickers in personal communication messages
Source: F1000Res. 2022 Aug 9;11:915. [Version 1] doi: 10.12688/f1000research.122623.1 (PMC9428498; doi:10.12688/f1000research.122623.1)
Supplement: Peer review history

# Peer review: Reviewer response for version 1

**DOI:** 10.5256/f1000research.134641.r146999

**Published:** 2022-08-30

**Authors:** William Philip Wall

**Affiliations:** 1 Faculty of Business and Technology, Stamford International University, Bangkok, Thailand

The article investigates the personality traits and engagement factors affecting the use of paid stickers in messaging. Based on the research study, the use of paid stickers in communication messages significantly influenced by perceived enjoyment and perceived ease of use. 

 Authors have used multiple theories including Technology Acceptance Theory (TAM) and Big Five Personality Traits to develop the research framework and conducted an empirical analysis. 

 Author has comprehensively covered the background and literature review section. 

 Analysis have been performed using Reliability, Validity, Confirmatory Factor Analysis and Structural Equation model. The indices of all the models shows the excellent fit. 

 With my expertise in the business management discipline, I would like to endorse my recommendation to consider its indexation; however, some of the optional improvements are recommended which can be considered by Author. 

**Main comments:**

 In the article, there are no directions of future research for the other researchers to contribute their results from different environments.   

 The article has comprehensively covered the implications and contributions, and conclusion section which gives a complete visibility to its readers about the importance of paid stickers in communication messages. 

 The respondent’s data furnished along with the article testifies the study conducted in authentic, and legit. 

 Also, the results, and discussion section provides the comprehensive information about the article’s findings and relating the results from the international studies.****

**Specific comments**

 Author have detailed the information about the introduction and background of the topic. Also, detailing about the problems of the research study has made it convenient for readers easy understanding.  

 Author can also consider incorporating the research objectives to bring it more clarity.  

 The article has comprehensively interpreted the results.  

 The discussion section has comprehensively covered the information relating it with previously conducted research studies and also studies conducted internationally highlighting the findings. 

 Also, the tabulated illustration of research findings provides its readers an opportunity to determine the hypotheses tested significant.

Is the work clearly and accurately presented and does it cite the current literature?

Yes

If applicable, is the statistical analysis and its interpretation appropriate?

Yes

Are all the source data underlying the results available to ensure full reproducibility?

Yes

Is the study design appropriate and is the work technically sound?

Yes

Are the conclusions drawn adequately supported by the results?

Yes

Are sufficient details of methods and analysis provided to allow replication by others?

Yes

Reviewer Expertise:

Business Management, Strategic Management, Entrepreneurship, Startups, Angel Investment, Renewable Energy, Sustainable Development.

I confirm that I have read this submission and believe that I have an appropriate level of expertise to confirm that it is of an acceptable scientific standard.

# Peer review: Reviewer response for version 1

**DOI:** 10.5256/f1000research.134641.r146997

**Published:** 2022-08-26

**Authors:** Babita Singla

**Affiliations:** 1 Chitkara Business School, Chitkara University, Rajpura, Punjab, India

I have reviewed this article in detail. It investigates the personality traits and engagement factors that affect the use of paid stickers in personal communication. The factors researched included conscientiousness, extraversion, openness, neuroticism, and ease of use. The use of paid stickers in personal communication was found to be majorly influenced by perceived ease of use and perceived enjoyment. 

 In this report, the author adopted the Technology Acceptance Model (TAM) and the Theory of big five personality traits and developed the conceptual framework and hypothesis on its basis. The theoretical background and empirical literature have been well established. 

 The author used structural equation modeling (SEM) with satisfactory results on the measurement model – including reliability, validity, and goodness of fit tests. 

 From a critical review of the article, I am of the view that the manuscript satisfies the required threshold for publication. However, I have several comments have been made regarding this manuscript, and have been summarized below. These comments do not require revisions, and the article can be indexed in its current form.

 Main comments:

- The article has adequately discussed the limitations of the study. However, it has not given a direction for future research. I believe that based on these limitations, the article could have highlighted the recommendations that could be considered by future researchers.
- The article has a major strength in that it has attached the data and model used. Therefore, the findings of the research are verifiable, and the reliability and validity examined
- Another notable aspect is that references, almost all of them are recent ones (previous five years). This is very commendable as it displays the time relevance of the article's findings 

Specific comments 

- In the introduction, the background of the topic is well introduced. As well, I was able to clearly identify the problem of the study. 
- However, I did not find a statement that clearly states the objective/purpose of the study. It is always advisable to state the objective of the study in addition to the problem statement  
- The results presentation is empirically satisfactory
- The discussion of the results has been done adequately. The findings of the article were compared with previous findings, with adequate citations. I find this commendable 
- For the model measurement, it is usually a good practice to present the findings of the model fitness on a table, alongside their threshold, and a column indicating whether the threshold has been achieved or not

Is the work clearly and accurately presented and does it cite the current literature?

Yes

If applicable, is the statistical analysis and its interpretation appropriate?

Yes

Are all the source data underlying the results available to ensure full reproducibility?

Yes

Is the study design appropriate and is the work technically sound?

Yes

Are the conclusions drawn adequately supported by the results?

Yes

Are sufficient details of methods and analysis provided to allow replication by others?

Yes

Reviewer Expertise:

Marketing and Retail

I confirm that I have read this submission and believe that I have an appropriate level of expertise to confirm that it is of an acceptable scientific standard.
